# Major depressive disorder in a Kenyan youth sample: relationship with parenting behavior and parental psychiatric disorders

**DOI:** 10.1186/1744-859X-12-15

**Published:** 2013-05-10

**Authors:** Lincoln I Khasakhala, David Musyimi Ndetei, Muthoni Mathai, Valerie Harder

**Affiliations:** 1 Department of Psychiatry, University of Nairobi, P.O. Box 59176 00200, Nairobi, Kenya; 2 Africa Mental Health Foundation, 1st Floor Gakuo Court, Lower Hill Road, Off Haile Sellasie Avenue, P.O. Box 4842300100, Nairobi, Kenya; 3 University of Nairobi, P. O. Box 30197, G.P.O., Nairobi, Kenya; 4 Department of Psychiatry, University of Vermont, 85 S Prospect St, Burlington, VT, 05401, USA

**Keywords:** Depressive disorder, Youth, Maternal depressive disorder, Perceived parental behavior, Co-morbid psychiatric disorders

## Abstract

**Background:**

Studies on mental health problems during childhood and youth development phases have reported that families of children diagnosed with a depressive disorder tend to be dysfunctional. These dysfunctions have been shown to be mediating factors for children to develop psychiatric disorders in the future.

**Objective:**

This study was designed to investigate whether perceived parenting behavior and parental psychiatric disorders have any relationship with youth presenting with major depressive disorder.

**Methodology:**

The study sample had a total number of 250 purposely selected youth attending the Youth Clinic at Kenyatta National Hospital in Nairobi.

**Results:**

This study found associations between major depressive disorders (MDD) in the youth and co-morbid psychiatric disorders among the youth: conduct disorder (OR = 2.93, 95% CI 1.04 to 8.26, *p* = 0.035), any anxiety disorder (OR = 2.41, 95% CI 1.20 to 4.87, *p* = 0.012), drug abuse (OR = 3.40, 95% CI 2.01 to 5.76, *p* < 0.001), alcohol use (OR = 3.29, 95% CI 1.94 to 5.57, *p* < 0.001), and suicidal behavior (OR = 5.27, 95% CI 2.39 to 11.66, *p* < 0.001). The results also indicate that a higher proportion of youth between 16 and 18 years had major depressive disorder than the youth below 16 years or above 18 years of age (OR = 2.66, 95% CI 1.40 to 5.05, *p* = 0.003). Multivariate analysis shows that both rejecting maternal behavior (AOR = 2.165, 95% CI 1.060 to 4.422, *p* = 0.003) and maternal MDD (AOR = 5.27, 95% CI 1.10 to 14.76, *p* < 0.001) are associated with MDD in youth.

**Conclusion:**

Negative maternal parenting behavior and maternal depressive disorder are associated with major depressive disorder in children.

## Background

Studies on mental health problems during child and youth development phases indicate that families of children diagnosed with a depressive disorder tend to be dysfunctional [1-7]. The dysfunctions include unhealthy quality of marital interactions, presence of psychiatric disorders among parents, and maladaptive parental behaviors. These dysfunctions have been shown to be mediating factors for children to develop psychiatric disorders [8-15]. It has also been shown that in this type of family setting, there exists a high rate of parent-to-parent or parent-to-child conflicts that make members highly vulnerable to develop a psychiatric disorder [1-15]. Previous studies in this area also show that youths brought up in these homes where one psychiatric disorder occurs often have one or more co-morbid psychiatric disorders [16-21]. This effect seems to be stronger when both parents suffer from any psychiatric disorder [8-15]. Compared to depressed youths of non-depressed parents, youths with a family history of depression have been found to suffer more severe and chronic forms of depression, more relapses, psychiatric co-morbidity, impaired psychosocial functioning, and suicidal behavior [21-23].

World Health Organization (WHO)-based studies indicate that the prevalence of psychiatric disorders previously seen in adult life has increased enormously among children and youths in the past few years [24-27]. Four percent of 12- to 17-year olds and 9% of 18- to 24-year olds have been shown to suffer from major depressive disorder (MDD) [26,27]. These WHO studies also indicate that depression is the most prevalent disorder worldwide with wide reaching consequences in youths [24-26]. Further, it has been shown that some forms of parenting styles are associated with child abuse. A study by Khasakhala et al. [28] found that there were statistical differences (*p* < 0.05) between perceived paternal permissive parenting behavior and emotional and physical neglect of the youths, perceived paternal authoritarian parenting behavior and emotional and physical abuse of the youths, while perceived uninvolved parenting behavior was found to be associated with both emotional and physical neglect of the youths. In the study, mothers who had authoritarian parenting style emotionally and physically abused their youths, while those who were uninvolved, emotionally and physically neglected their youths [28]. Uninvolved parents give negative attention to the behaviors and activities their youths display; this is the opposite of approval, and therefore, this behavior does not protect the youth from developing psychiatric disorders. Studies also indicate that untreated MDD in youths is associated with later development of anxiety disorders, bipolar mood disorders, and substance abuse disorders [18-20]. It has also been shown that youths with co-morbid depression come from dysfunctional families and have severe substance abuse disorders [8,10]. Depression has been shown to be associated with youth suicidal behavior which is a major problem in many countries, as it is the third leading cause of death in young people [11].

In Kenya, the prevalence of depression among youths attending general health facilities and those in secondary schools has been found to be high [8,10]. The prevalence of clinically depressive symptoms in Kenya is 43.7% among youths in public schools in Nairobi province, while the prevalence of those attending general health facilities is 41.3% [29,30]. Khasakhala et al. [28] found that more than a quarter of youths in high school suffer from MDD which has a relationship with aspects of perceived maladaptive parenting behavior. Parental psychopathology has also been shown to be associated with maladaptive parenting behavior. Thus, these family dysfunctions are characterized with poor communication, poor problem solving, and the presence of hostile criticism in the family [31-35]. Therefore, a parent with mental disorder has poor interactive skills perceived as a maladaptive parental behavior by children; this is a mediator for children to develop psychopathology, in particular MDD [31-35]. The nature of this association is of considerable interest to mental health workers and scientists alike, in part because it may be possible to reduce the odds that children will develop psychiatric disorders if parents are helped to modify their parental behavior or access psychiatric treatment in case the parent has a psychiatric disorder.

There is need therefore to document the negative and important roles that parents/caregivers play not only to help their children successfully transit into teenage/adulthood but also because this transition should have a healthy relationship void of psychopathology. The primary question addressed in this paper is whether the presence of parental psychiatric morbidity and perceived maladaptive parental behavior serve as useful indicators of associating MDD in youth age 13 to 25 years. The main aim of this paper was to assess the relationship between parents, psychiatric disorders and maladaptive parental behaviors and youths MDD. The hypothesis of the study was that parents with a psychiatric disorder face challenges in bonding with their children as they exhibit maladaptive parenting behavior, a mediator for their offspring's to develop psychiatric disorders. This study was designed to address the magnitude of the burden of disease related to psychiatric disorders in a family setting in Kenya, which remain unrecognized and undertreated. Studies carried out in developed countries indicate that parental psychopathology is associated with maladaptive parenting behavior [13,15,36-38], and maladaptive parenting behavior is associated with an increased likelihood of youths developing mood disorders [3-6].

## Method

### Participants

Participants in this study included 250 purposefully sampled youth attending the outpatient psychiatric clinic at Kenyatta National and referral Hospital (KNH) in Kenya. They were recruited into the study after psychological interview and were diagnosed with any DSM IV axis I psychiatric disorder including bipolar mood disorder, schizophrenia, post-traumatic stress disorder, any other anxiety disorder, substance use disorder, alcohol use disorder, and conduct disorder. The age range of the youths recruited was 13 to 22 years with a mean age of 16.92 years, median of 17 years, and standard deviation of 2.151. They were categorized into three groups: 13 to 15, 16 to 18, and 19 to 22 years.

The researcher, however, did not succeed in recruiting all parents, only 226 and 202 biological mothers and fathers, respectively, were reached. The youths were selected to participate in the study if they had a DSM-IV axis I psychiatric disorder, scored above 25 points on the Mini Mental State Examination (MMSE) and had at least one parent (biological) enrolled in the study.

### Procedure

Approval for data collection was obtained from KNH and the University of Nairobi Ethics and Review Board. Consent from every parent and youth above 17 years of age and parental consent for youths between ages 13 to 17 years who assented to participate were obtained prior to study participation. Among the excluded youths (5.7% (15)), six did not return to the clinic for follow-up despite several telephone reminders about their appointment dates, five parents did not sign the consent forms, and four youths had severe psychotic disorder and, therefore, did not meet the final criteria (scored less than 25 points on the Mini Mental State Examination).

### Measures

Closed-ended, face-to-face interviews were conducted with participants at KNH Youth Centre using a researcher-formulated socio-demographic, structured clinical interview schedule using Mini International Neuropsychiatric Interview for Children and Adolescents (MINI Kid) administered to the adolescents 13 to 17 years and Mini International Neuropsychiatric Interview for Adults (MINI Plus) to parents and youth above 17 years of age [39-41]. Both MINI Kid and MINI Plus are structured diagnostic interview schedules developed for the diagnoses of DSM-IV and ICD-10 psychiatric disorders [41,42]. These structured questionnaires are designed to meet the need for a short but accurate structured psychiatric interview for multi-center clinical trials and epidemiology studies. These schedules were used in this study as a first step in outcome tracking and confirming the axis I DSM-IV disorders. The interview questions are designed to elicit specific diagnostic criteria according to DSM-IV diagnosis [41,43].

The MMSE, a most commonly used test for assessing memory and cognition problems, was filled for all respondents [44]. In this study, the MMSE was used to screen for the presence of cognitive impairment on mental activities such as memory, thinking, calculation, language, constructional ability, reasoning, decision making, orientation in time and place, attention, immediate and recall memories, and dealing with concepts, i.e., abstraction. Developed by Dr. Marshal Folstein in the 1970s [44], the MMSE has been used not only as a clinical tool but also as a research tool in developed countries such as UK and USA and in developing counties including Kenya, Ecuador, Uganda, and South Africa. It has been translated into over 50 languages, and it is a very useful broad screening test, especially when it is suspected that mental functions are severely compromised.

Perceived parental behavior by youth in the study was assessed using the Egna Minnen Betraffande Uppfostran (EMBU) questionnaire, in English as ‘own memories of childhood upbringing’ [45-47]. This is a self-administered questionnaire about perceived parental behavior. Youths were asked to recall in what way their parents were alike and in what way the parents differed using the questionnaire that has 81 items. In each question, the youths considered how their father behaved and then how their mother behaved towards them. The 81 items in the questionnaire measure two constructs that has a total of eight factors, four parenting styles and forms of child abuse which are further computed into four types of parental behaviors [45]. Parenting styles include authoritative, authoritarian, permissive, and uninvolved, while the forms of child abuse include emotional abuse, emotional neglect, physical abuse, and physical neglect. The four types of parental behavior are the following: (1) no emotional attachment computed from two forms of child abuse (emotional abuse and physical abuse) and the authoritarian parenting style, (2) rejecting parental behavior computed from both emotional and physical child neglect, (3) under-protective parental behavior computed from both permissive and uninvolved parenting style, and (4) authoritative parental behavior from the computation of authoritative parenting style.

The socio-demographic questionnaire was filled in the presence of both youth and parent(s). The structured psychiatric interview schedule and MMSE for each participant were conducted confidentially on one-to-one but later were matched. The researcher received intensive training on the administration of structured interview questionnaires both MINI Kid and MINI Plus. Commitment checks were regularly carried out for youth and their parents to ensure that the study protocol was adhered to. Interviews lasted approximately 50 to 60 min. Each participant was first screened for any psychiatric disorder using MINI Kid or MINI Plus screen. The participants were asked if they had any specific feeling or behavior in the past on the screener, and the response was either ‘yes’ or ‘no’. For items on the screener with a ‘yes’, the participants were further interviewed to make a specific DSM-IV-TR diagnosis using MINI Kid or MINI Plus main questionnaire. These questionnaires (MINI Kid and MINI Plus) have high reliability and validity and been adapted and translated into Kiswahili in the East African region [40]. The youths filled out the self-administered EMBU questionnaire, where they rated perceived specified parenting behavior of each parent since childhood on a Likert scale from 1 (0 as no never) to 4 (3 as yes always) [45-47]. Higher scores on the three types of parenting behavior indicated maladaptive parental behavior, while high score on perceived authoritative parenting behavior was considered adaptive parental behavior.

### Statistical analysis

Data analysis using SPSS version 16 to describe each DSM-IV diagnosis (multiple) of each participant was done by summing up the ‘yes’ responses that met each criterion for DSM-IV I disorder. A chi-square test was run to assess the association of covariates, both the socio-demographic data and psychiatric disorder among the youths and their parents separately. Using the chi-square test, further associations between perceived parenting behavior and depressive disorder were assessed. Lastly, multiple logistic regression of maternal depression and parenting behavioral problems was used to assess the association with youth MDD, controlling for age and gender. Given the large number of repeated chi-square tests, the *p* value was set at 0.01 based on a conservative Bonferroni criterion.

## Results

A total of 250 youths were recruited, but only 245 were included in the final analysis. Five youths declined to allow their parents to participate as they were (sexually) abused in a family setting.

### Factors associated with major depressive disorders (MDD) in youths

#### Bivariate analyses

Table 1 presents the analysis of MDD among youths in relation to background characteristics. The occurrence of MDD was associated with late adolescence, 16 to 18 years (OR = 2.66, 95% CI 1.40 to 5.05, *p* = 0.003), but marginally associated with younger adolescents 13 to 15 years (OR = 1.93, 95% CI 0.93 to 4.01, *p* = 0.078) compared to the age category of 19 to 22 years.

**Table 1:** Major depressive disorder among youth in relation to socio-demographic characteristics and duration of mental disorder

**Variables**	**Present (*N *= 133)**		**Absent (*N *= 112)**		**OR**	**95% CI**		***p *Value**
	***n***	**%**	***n***	**%**		**Lower**	**Upper**	
Age in years								
13 to 15	33	54.1	28	45.9	1.93	0.93	4.01	0.078
16 to 18	78	61.9	48	38.1	2.66	1.4	5.05	*0.003*
19 to 22	22	37.9	36	62.1	Reference			
Sex								
Female	53	53	47	47	0.92	0.55	1.53	0.737
Male	80	55.2	65	44.8	Reference			
Position of birth								
Only child/first born	68	61.3	43	38.7	1.74	0.68	4.44	0.247
Second born	37	67.3	18	32.7	2.26	0.81	6.3	0.119
Third born	10	27.8	26	72.2	0.42	0.14	1.3	0.134
Fourth born	8	36.4	14	63.6	0.63	0.19	2.13	0.456
Fifth born or higher	10	47.6	11	52.4	Reference			
Level of education								
Primary	37	54.4	31	45.6	2.03	0.81	5.07	0.130
Secondary	86	57.3	64	42.7	2.28	0.98	5.32	0.055
College	10	37.0	17	63.0	Reference			
Marital status of parents								
Single mother, never married	7	43.8	9	56.3	0.68	0.24	1.9	0.463
Widower/widow	22	55.0	18	45.0	1.07	0.54	2.13	0.852
Orphan	6	100.0	0	0.0	UD	UD	UD	0.999
Separated	3	60.0	2	40.0	1.31	0.21	8.03	0.770
Married	95	53.4	83	46.6	Reference			
Duration of mental disorder								
1 to 6 months	15	53.6	13	46.4	1.15	0.27	4.89	0.846
7 to 12 months	23	50.0	23	50.0	1.00	0.25	3.93	1.000
>1 to 2 years	40	57.1	30	42.9	1.33	0.35	5.03	0.671
>2 to 5 years	28	48.3	30	51.7	0.93	0.24	3.57	0.920
>5 to 10 years	16	59.3	11	40.7	1.45	0.34	6.25	0.614
>10 years	5	50.0	5	50.0	Reference			
Unknown	6		0					

The italicized value is at *p* < 0.05. CI, confidence interval; OR, odds ratio; UD, undefined.

Table 2 presents the analyses of MDD among the youths in relation to mental health status of the parents. Under-protective behavior of fathers was associated with reduced cases of MDD among the youth (OR = 0.16, 95% CI 0.04 to 0.72, *p* = 0.017). The occurrence of MDD among the youths was associated with death of the fathers with reference to the absence of MDD in fathers (OR = 2.24, 95% CI 1.12 to 4.51, *p* = 0.023).

**Table 2:** Major depressive disorder among youths in relation to mental health status of the parents

**Variables**	**Present (*N *= 133)**		**Absent (*N *= 112)**		**OR**	**95% CI**		***p *Value**
	***n***	**%**	***n***	**%**		**Lower**	**Upper**	
Behavior of mothers								
Not emotional	9	32.1	19	67.9	0.71	0.21	2.35	0.575
Rejecting	81	62.8	48	37.2	2.53	0.97	6.63	0.059
Under protective	25	46.3	29	53.7	1.29	0.46	3.67	0.629
Normal	8	40.0	12	60.0	Reference			
Unknown	10		4					
DD in mothers								
Present	93	57.1	70	42.9	2.14	0.68	3.93	0.163
Absent	28	50.9	27	49.1	Reference			
Deceased	12	70.6	5	29.4	2.27	0.75	6.88	0.146
Behavior of fathers								
Normal	17	63.0	10	37.0	Reference			
Not emotional	60	57.7	44	42.3	0.80	0.34	1.92	0.620
Rejecting	26	53.1	23	46.9	0.66	0.25	1.74	0.406
Under protective	3	21.4	11	78.6	0.16	0.04	0.72	*0.017*
Unknown	27		24					
MDD in fathers								
Present	18	47.4	20	52.6	0.86	0.42	1.74	0.667
Absent	82	51.3	78	48.8	Reference			
Deceased	33	70.2	14	29.8	2.24	1.12	4.51	*0.023*
Alcohol use among fathers								
Present	50	52.1	46	47.9	1.13	0.65	1.97	0.667
Absent	50	49.0	52	51.0	Reference			
Deceased	33	70.2	14	29.8	2.45	1.17	5.12	*0.017*

Italicized values are at *p* < 0.05. CI, confidence interval; OR, odds ratio.

Chi-squire test of MDD among youths in relation to other mental health status of the youths are presented in Table 3. MDD was associated with conduct disorder, any anxiety disorder, any drug abuse problem, and alcohol use disorders.

**Table 3:** Major depressive disorder among youths in relation to other psychiatric disorders

**Variables**	**MDD (*N *= 133)**		**No MDD (*N *= 112)**		**Total (*N *= 245)**	***p *Value**
	***n***	**%**	***n***	**%**	***n *(%)**	
Conduct disorder						
Yes	16	76.2	5	23.8	21 (8.6)	*0.045*
No	117	52.2	107	47.8	224 (91.4)	
Any anxiety disorder						
Yes	32	71.1	13	28.9	47 (19.2)	*0.015*
No	101	50.5	99	49.5	198 (80.8)	
Any drug abuse						
Yes	112	73.2	41	26.8	153 (62.4)	*<0.001*
No	21	22.8	71	77.2	92 (37.6)	
Alcohol abuse						
Yes	81	69.2	36	30.8	117 (47.8)	*<0.001*
No	52	40.6	76	59.4	128 (52.2)	
Suicide behavior						
Yes	124	60.5	81	39.5	205 (83.8)	*<0.001*
No	9	22.5	31	77.5	40 (16.2)	

Italicized values are at *p* < 0.05.

The analyses of MDD among youths in relation to other mental health status of the youths are presented in Table 4. The occurrence of MDD was associated with conduct disorder (OR = 2.93, 95% CI 1.04 to 8.26, *p* = 0.035), any anxiety disorder (OR = 2.41, 95% CI 1.20 to 4.87, *p* = 0.012), any drug abuse (OR = 3.40, 95% CI 2.01 to 5.76, *p* < 0.001), alcohol use (OR = 3.29, 95% CI 1.94 to 5.57, *p* < 0.001), and suicidal behavior (OR = 5.27, 95% CI 2.39 to 11.66, *p* < 0.001).

**Table 4:** Major depressive disorder among youths in relation to other mental health status of the youths

**Variables**	**Present (*N *= 133)**		**Absent (*N *= 112)**		**OR**	**95% CI**		***p *Value**
	***n***	**%**	***n***	**%**		**Lower**	**Upper**	
Conduct disorder								
Yes	16	76.2	5	23.8	2.93	1.04	8.26	0.035
No	117	52.2	107	47.8	Reference			
Any anxiety disorder								
Yes	32	71.1	13	28.9	2.41	1.2	4.87	*0.012*
No	101	50.5	99	49.5	Reference			
Any drug abuse								
Yes	87	68.5	40	31.5	3.40	2.01	5.76	*<0.001*
No	46	39.0	72	61.0	Reference			
Alcohol use								
Yes	81	69.2	36	30.8	3.29	1.94	5.57	*<0.001*
No	52	40.6	76	59.4	Reference			
Suicide behavior								
Yes	124	60.5	81	39.5	5.27	2.39	11.66	*<0.001*
No	9	22.5	31	77.5	Reference			

Italicized values are at *p* < 0.05. CI, confidence interval; OR, odds ratio.

### Multivariable analyses

Multiple logistic regressions were used to model the occurrence of MDD using factors during bivariate analyses, as tabulated in Table 5. Eight independent indicator variables of MDD among the youth were identified. Adjusting for other factors, age category 16 to 18 years was associated with occurrence of MDD (AOR = 2.74, 95% CI 1.09 to 6.93, *p* = 0.033). Rejecting maternal behavior was identified to be associated with MDD in youth (AOR = 2.165, 95% CI 1.060 to 4.422, *p* = 0.034). The occurrence of MDD in mother was identified to be associated with MDD in youth (AOR = 5.27, 95% CI 1.10 to 14.76, *p* < 0.001). The occurrence of MDD in father was not identified to be associated with MDD in youth (AOR = 0.51, 95% CI 0.21 to 1.23, *p* = 0. 135). However, death of the father was identified to be associated with MDD among the youths (AOR = 4.69, 95% CI 1.50 to 14.69, *p* = 0.008). Other mental health disorders of the youths were also identified to associated with MDD, which included any anxiety (AOR = 4.03, 95% CI 1.47 to 11.08, *p* = 0.007), alcohol use (AOR = 2.41, 95% CI 1.19 to 4.89, *p* = 0.015), and suicidal behavior (AOR = 4.52, 95% CI 1.38 to 14.81, *p* = 0.013).

**Table 5:** Adjusted odds ratios of major depressive disorder among Kenyan youth

**Associations**	**AOR**	**95% CI**		***p *Value**
		**Lower**	**Upper**	
Age in years				
13 to 15	1.64	0.58	4.60	0.348
16 to 18	2.74	1.09	6.93	*0.033*
19 to 22	Reference			
Gender	1.98	0.87	8.678	*0.045*
Female	Reference			
Male				
Maternal parental behavior				
Rejecting behavior	2.165	1.060	4.422	*0.034*
Other behavior	Reference			
Major depressive disorder in mother				
Present	5.27	1.10	14.76	*<0.001*
Absent	Reference			
Major depressive disorder in father				
Present	0.51	0.21	1.23	0.135
Absent	Reference			
Deceased	4.69	1.50	14.69	*0.008*
Any anxiety in youth				
Yes	4.03	1.47	11.08	*0.007*
No	Reference			
Alcohol use in youth				
Yes	2.41	1.19	4.89	*0.015*
No	Reference			
Suicide behavior in youth				
Yes	4.52	1.38	14.81	*0.013*
No	Reference			
Conduct disorder in youth				
Yes	2.909	0.659	12.848	*0.159*
No	Reference			

Italicized values are at *p* < 0.05. AOR, adjusted odds ratio; CI, confidence interval.

## Discussion

Our main findings indicate that Kenyan youths with MDD seeking mental health treatment services are more likely to have mothers with MDD and are more likely to perceive their mothers as exhibiting a rejecting parenting behavior. This finding is similar to previous studies which have shown that the presence of psychiatric disorders among parents, which is associated with maladaptive parental behaviors, is a mediating factor for children to develop psychiatric disorders [8-15]. Focusing first on the intergenerational associations of MDD, our findings are comparable to studies that use the ‘top-down’ approach which consistently shows that children of depressed parents have a substantially increased risk to experience not only depressive disorders but also other DSM-IV disorders [1,2,4-11]. These results are also comparable to ‘bottom-up’ studies that examine clinically referred depressed children and adolescents, which showed increased rates of depression and other forms of psychopathology in parents [19-23]. Although perceived parenting behavior models play different roles for different youth psychosocial outcomes, overall, our results support the attachment theory which highlights the importance of specific perceived parenting behavior, the attachment between a child and their parents. Our findings are consistent with the notion that parental depression has a negative impact on the emotional and behavioral functioning of offspring. This is because parental depression leads to family disruption and marital discord which has a negative impact to the mental well-being of children.

Our study found that youths with MDD also were more likely to have other co-existing DSM-IV axis I disorders (substance abuse, any anxiety, and conduct disorder), suggesting that co-morbid psychiatric disorders also need attention during treatment. The perceived rejecting maternal behavior as shown in this study is a negative factor inducing severe psychological distress; therefore, a child with depressive disorder tries to escape from internalizing their feelings (depression) by acting out (externalizing them) and, in the process, starts abusing alcohol/substances. This is a similar finding by Rankin et al. [48]. This raises the possibility that parental behavior may be a risk factor in the development of multiple psychiatric disorders in the same child.

The result in this study indicate that multiple independent variables associated with MDD in youth include the following: rejecting maternal parenting behavior, maternal MDD, and other co-morbid psychiatric disorders among youths. Rejecting maternal parenting behavior and depressive disorder in a parent are associated with greater odds for children to develop MDD. Rejecting behavior plays an important role in the development of psychiatric disorders in children, and this had an association with parents' psychiatric disorders. This finding may explain the dysfunctional family nature in this study population. Parental psychopathology, in particular depressive disorder in mothers, could have a paring to genetic predisposition which in turn is associated with greater odds for children to develop depressive disorder. This explains how disconnected this family setting functions. There is increased conflict in this type of family setting. The perceived maternal rejecting parenting behavior obstructs the interaction between mother and her children. The rejecting parenting behavior in such a family setting is perceived by children to be a poor emotional expression, ‘I have no interest in you’ and therefore disconnects the child from the mother, creating a barrier for the child to explore and form connecting bonds with his/her parent(s). This barrier results into insecure attachment which was described earlier by Bowlby [49-51]. This unconnectedness between the child and parent leads to confusion, conflict, and frustration in the growing child, a precursor for a child to develop psychopathology which presents as either an internalizing (depression/anxiety) or externalizing disorder (alcohol abuse/conduct).

As indicted by the results of this study, a high proportion of youth who perceived that their mother had rejecting parenting behavior had higher odds of developing depression and abusing alcohol/substance (multiple substances). This finding is comparable to prior family studies in patient samples [31-34], which revealed that parental psychopathology is associated with maladaptive parental behavior and, in turn, is associated with increased odds of psychopathology among their children. This demonstrates that children who perceive their mothers to have rejecting parental behavior are more likely to develop MDD than children of parents with other parental behavior.

Previous studies [31-34] suggest potential explanations for how parental characteristics may contribute to MDD in their children. Rejecting maternal behavior may restrict the child's development of autonomy that leads to competence which allows the developing child to explore their environment. Perceived parenting rejection by mothers may lead to a dysfunctional parent–child bond, which may result in difficulties for the child to explore the environment, leading to a helplessness experience. In addition, rejection may keep the child from engaging in social situations, thereby restricting the opportunities to learn social skills and therefore remain inferior. These findings are of interest, in particular, if the onset of mental disorders among youth can be prevented as suggested by Bowlby [49-51], whereby parents can be assisted to modify their child-rearing practices.

More importantly, from these results, rejecting maternal parenting behavior may play a role in the development of psychopathology in children whether or not a mother has psychopathology. This is more so because rejecting maternal parenting behavior is relatively more common in our society [45]; therefore, it may be important to educate the public about these abnormal parenting styles that are associated with an increased risk of offspring to develop psychopathology. This data is consistent with previous research findings which have indicated that parental psychopathology is associated with maladaptive parental behavior [11,29-32] which is also associated with increased risk of the children to develop psychopathology. Perceived rejecting maternal parental behavior was the only independent variable, where higher scores on the rejecting-ineffective parenting scale were associated with higher odds of MDD among the youth. Perceived rejecting maternal parenting behavior therefore influences family life, and parental psychopathology is linked to poor child-rearing practices. This social life around the growing child defines important tasks that the growing person needs to achieve.

### Limitations

While interpreting the results of this study, three paramount limitations should be taken into account. First, the cross-sectional nature of the study limited the ability to make inferences about causality. We cannot be sure whether parents were connected to their children, because majority had psychopathology and abnormal parenting behavior which are associated with youth negative psychological outcomes (psychiatric disorder). Nevertheless, we did control for several potentially spurious variables (age, other psychiatric disorders among youth, and parental psychopathology/parenting behavior) that helped strain the relationship between parents as role models and youth psychiatric disorders. Future research that uses longitudinal designs can help address this issue.

A second limitation of this study is reliance on self-report data by youth on perceived parenting behavior. This assessment measure did not provide more detailed information about the parent–child relationship. Additional information on the nature or quality of the relationship parents have with their children would help provide a clearer picture of how parents with or without a psychiatric disorder influence youth to develop a psychiatric disorder. This information would allow studying the potential effects of parenting qualities. Nevertheless, our study suggests that parenting behavior as perceived by children, and parental psychiatric disorders have a powerful influence on children to develop psychiatric disorder. Hence, continued research to further understand this relationship is warranted.

Third, this study was done in Kenya, where mental health services are scarce and inaccessible. Therefore, when interpreting these results, it is important to consider that the respondents might not have understood the meaning of psychiatric disorders. This is because, in most communities, the concept of mental health as defined in the western countries has been fully developed in the Kenyan (African) context.

## Conclusion

These results provide vital insights into the intergenerational effects on child mental health. The study adds to the existing body of research on the role of parenting behavior and parental psychiatric disorders and their associations with youth MDD. Our main findings indicate that perceived rejecting maternal parenting behavior and maternal MDD are associated with youth MDD.

## Competing interest

This work is based on a PhD study by the first author. The authors declare that they have no competing interests.
